# Editorial: Translational research advancements utilizing the Iowa Gambling Task in preclinical and clinical studies: 30 years of the IGT

**DOI:** 10.3389/fpsyt.2025.1686127

**Published:** 2025-09-08

**Authors:** Vicki A. Nejtek, Michael F. Salvatore

**Affiliations:** ^1^ College of Biomedical and Translational Sciences, University of North Texas Health Science Center, Fort Worth, TX, United States; ^2^ The Parkinson Discovery Institute, Fort Worth, TX, United States

**Keywords:** translational, Iowa Gambling Task, IGT, evidence-based data, cross-species, pre-clinical

Merging outcomes from rodent and human studies is critical to identify underlying mechanisms of behavior in health and disease. Critical elements needed in experimental design to optimize translation of animal study results have been identified and proposed (1, 2). However, there remains an insufficient level of awareness of this perspective that has generated unwarranted skepticism that hinders necessary evolutionary growth of translational research. This special 30^th^ Anniversary Research Topic of the Iowa Gambling Task (IGT) features original studies and comprehensive reviews that rigorously challenge perceptions that cross-species translational research has limited reproducibility or generalizability to clinical populations. Since Bechara's seminal 1994 publication (3), the IGT has provided crucial insights on brain networks involved in cognitive processing (4–7) influencing decision-making (4, 8), revealing those at-risk for poor health trajectories in addiction (9–13), impulsivity (14), psychiatric illnesses (13–15) and neurodegenerative disease (16, 17).

Rodent studies, built upon translatable experimental designs, lend themselves well to elucidate neurobiological mechanisms (16, 18), and the rodent IGT (rGT) represents such an advancement for insight into mechanisms of decision-making in humans (19–21). Preclinical data from rGT studies must be recognized as the missing link to reveal therapeutic drug or gene targets that, in this case, will improve strategic decision-making in diseases where executive function is vulnerable (9–13, 15, 16, 22). Such cross-species translational studies provide critical evidence-based data needed to improve our ability to identify individuals at-risk for severe pathology or disability.

Cross-species translational research has shed light on the influence of human realities of aging, biological sex, psychological stress, and neurobiological perturbations on cognitive function. Singh et al. provide an unprecedented wealth of comprehensive data from rat (N = 170) and human studies (N = 722), illustrating many strong correlations between species and similar differences. For instance, in contrast to men, women make better decisions when there are fewer risks and punishments. The underlying neurobiological mechanisms of these biological sex-related and stress-induced differences are being identified with rGT paradigms. Their review elegantly aligns the preclinical and clinical paradigms and results, defines variables of cross-species alignment in behavioral patterns, and highlights relevant neurocognitive brain areas driving these variables.

Pratt and Morris recommend three significant factors to align neurocognitive processes between humans and rodents, thereby ensuring cross-species face, construct, and predictive validity: (1) the rGT paradigm should interrogate the same neural circuitry of humans, (2) the neurocognitive domain evaluated is comparable in both species, and (3) the behavioral constructs (i.e. stress, impulsivity, etc.) elicited between species align. This incisive review chronicles the earliest and most recently refined rGT protocol to generate translational data that satisfy the rules of face, construct, and predictive validity. Finally, they discuss how genetic and neural circuitry manipulation, environment, and age affect the rodent’s decision-making process that maps neatly onto human cognitive processes in psychiatric illnesses.

Rehn et al. focus on gene and environmental interactions impacting decision-making by investigating polymorphisms in genes that regulate monoamines, a serotonin transporter gene and monoamine oxidase A. Those carrying the short (S) allele in serotonin transporter gene and MAOA confers less transcription efficiency, decreasing serotonin reuptake and monoamine metabolism, which increases risk of impulsivity, gambling, and response inhibition. They evaluated the impact a negative vs. positive parenting environment had on IGT performance in S-allele carriers (age 18 - 22). Individuals living with negative parenting had lower IGT scores, whereas individuals living with positive parenting had the highest IGT scores, despite both groups carrying the same S-alleles. These differences were restricted to males, adding to the evidence of sex-based differences in decision-making. This study makes a compelling case for gene-environment interaction and impact on decision-making capabilities, setting the stage for strategic mechanistic preclinical research.

Salice et al. scoping review evaluates 7 studies on transcranial direct current stimulation (tDCS) impact on IGT performance in healthy individuals and those with clinical conditions. As tDCS evaluates active brain pathways during IGT performance, they summarize evidence that dorsolateral prefrontal cortex (DLPFC) and orbitofrontal cortex (OFC) are active in decision-making and compare differences between decisions involving risks versus those that are ambiguous (unknown options for risks). They conclude that tDCS enhances IGT performance in healthy adults and in patients with 3 different conditions that affect decision-making, including Parkinson’s disease. Stimulating DLPFC enhances goal-directed decision-making that involves risk assessment, whereas OFC could be targeted to improve feedback learning to inhibit disadvantageous choices. Thus, IGT-based therapies targeting DLPFC and/or OFC may improve decision-making associated with medication compliance vs. non-compliance in individuals with neurological or psychiatric disorders (16).

**I**ndividual differences in focusing attention, efficient working memory, and IQ take center-stage in Orm et al. In schizophrenia spectrum disorders, researchers reported IQ and executive function affected IGT performance, with lower IQ leading to disadvantageous decision-making. Going forward, they conclude low-IQ individuals with schizophrenia can benefit from interventions to mitigate the impact of impaired decision-making. They proposed that new cognitive assessments should be developed, because cortical networks are compromised in schizophrenia, thereby confounding IGT data interpretation. Similarly, variability in rat training time with the rGT has been speculated as a confound to rGT data interpretation. However, Lindberg et al. exquisitely addressed this long-standing question by scrutinizing training effects on learning speed and found time-to-train did not affect decision-making strategies or behavioral profiles. Their results are consistent with outcomes reported in a double-blind randomized clinical intervention trial, wherein despite monthly testing, learning the IGT strategy rarely occurred in subjects with bipolar disorder, comorbid with stimulant dependence (13).

Latibeaudière et al. provides comprehensive insight concerning IGT’s ability to assess the variability of anticipation, choices, and feedback that either enhances or degrades learning processes involved in decision-making. Here, event-related potentials (ERPs) obtained during the IGT reveal unique neural activation associated with novel decision-making that can characterize distinct neuropathologies such as Multiple Sclerosis, Borderline Personality Disorder, and Parkinson’s disease (PD). Doshier et al. reported in PD patients that poor IGT performance coincided with poorer performance on cognitive tests of executive function. Currently, PD is diagnosed after motor impairment, at which time major neuron loss has already occurred. Thus, detecting PD at the earliest pre-motor stage is critical. As impaired cognitive function can occur 10 years prior to diagnosis (22), the IGT could serve as a first line diagnostic tool to identify PD before motor impairment, giving clinicians a chance to forestall disease progression with preventative therapies.

In summary, evidence clearly shows that the IGT and rGT are invaluable in numerous neurological and psychiatric disorders to acquire evidence, and the mechanistic basis thereof, of impaired cognitive functions leading to non-strategic decision-making. As impaired executive function is comorbid with several neurological and psychiatric conditions, the IGT is invaluable for detecting current or eventual impairments. With the rGT, the mechanistic basis for these deficiencies can be revealed, thus giving avenues to improve decision-making and thwart disease progression in vulnerable individuals.
